# Proceedings of the Galen Medical Society, of Stark Co., Ind.

**Published:** 1864-11

**Authors:** L. D. Glazebrook, J. B. Hoag

**Affiliations:** President; Secretary


### PROCEEDINGS OF THE GALEN MEDICAL SOCIETY, OF STARK CO., IND.

This Society was organized July 9th, 1864, and has held two meetings since its organization. At the last meeting, which was held Oct. 1st, 1864, the Secretary was instructed to furnish a synopsis of the proceedings of that and the previous meetings to the *Chicago Medical Journal* for publication.

The Society was organized at Knox, Ind., July 9th, 1864. Dr. L. D. Glazebrook, of Saint Pierre, was chosen President, and Dr. J. B. Hoag, of Knox, Secretary.

The principal features of that meeting were:

1st. Passing a preamble and resolutions setting forth that, in consequence of the advance in prices on flour, meat, groceries and dry goods, as well as a corresponding increase in the price of medicines, it was necessary for physicians to imitate "the rest of mankind" and give another turn to the crank of prices; and forming a fee-bill, in conformity with said resolutions, which was signed by all the members, who pledged themselves to be governed by it.

2nd. Passing a rule that each physician should furnish each other physician with a list of those who were indebted to them, and refused to liquidate their claim, but were able to do so; and pledging themselves not to administer to such persons or their families until their former physician's bills had been paid.

3rd. Passing a resolution expressive of their willingness to practice for indigent widows and orphans, and all other proper objects of charity, whether they were compensated or not.

4th. The framing of a Constitution by which the Society was to be governed was deferred until the next meeting.

5th. The Society adjourned, to meet at Knox, August 6th, 1864.

The second meeting of the Society was held at Knox, Aug. 6th, 1864, pursuant to adjournment.

The main features of this meeting were:

1st. Adopting a Constitution by which the Society is to be governed.

2nd. Requiring each member to report one or more cases at each meeting, giving diagnosis and treatment.

3rd. Making it the duty of the President to appoint, at each meeting, one, to prepare an essay on some subject of interest, to be read at the next meeting.

4th. Dr. J. B. Hoag was appointed to produce an essay to be read at the next meeting. Subject—The duties and responsibilities of the physician.

5th. The Society adjourned, to meet at San Pierre, Oct. 1st, 1864, at 2 o'clock P. M.

The Society met at San Pierre, Oct. 1st., pursuant to adjournment.

The principal features of the meeting were:

1st. It was voted that before names were reported as delinquents a settlement should be demanded of them, and they informed that they had to settle within a reasonable time to be designated.

2d. It was voted that the members of the Society refuse to counsel with an irregular physician, except in company with another regular physician.

3rd. Several cases, with diagnosis and treatment, were reported.

4th. Dr. J. B Hoag read an essay on the "Duties and responsibilities of the physician," which he was requested to forward to the *Chicago Medical Journal* for publication, with a synopsis of the proceedings of this and the former meetings of this Society.

5th. Dr. S. B. Coltett was appointed to prepare an essay to be read before our next meeting, the subject to be selected by himself.

6th. Adjourned, to meet at Knox, Nov. 5th, 1864, at 2 o'clock.

L. D. GLAZEBROOK, President.

J. B. HOAG, Secretary.

